# Supplementary material for: Association between Recent Usage of Antibiotics and Immunogenicity within Six Months after COVID-19 Vaccination
Source: Vaccines (Basel). 2022 Jul 14;10(7):1122. doi: 10.3390/vaccines10071122 (PMC9318721; doi:10.3390/vaccines10071122)
Supplement: Supplementary file 1 [file vaccines-10-01122-s001.zip › vaccines-1780900-supplementary.pdf]

**Table S1. Anti-bacterial spectrum of various classes of antibiotics**

| Class | Anti-bacterial spectrum | Route |
|---|---|---|
| ***Penicillins (n=26)*** | | |
| Benzylpenicillin | Narrow spectrum | Intravenous |
| Phenoxymethylpenicillin | Narrow spectrum | Oral |
| Benzathine penicillin G | Narrow spectrum | Intramuscular |
| Penicillin G procaine | Narrow spectrum | Intramuscular |
| Cloxacillin | Narrow spectrum | Oral |
| | Narrow spectrum | Intravenous |
| Flucloxacillin | Narrow spectrum | Oral |
| | Narrow spectrum | Intravenous |
| Amoxycillin +/- clavulanate | Broad spectrum | Oral |
| | Broad spectrum | Intravenous |
| Ampicillin +/- sulbactam | Broad spectrum | Oral |
| | Broad spectrum | Intravenous |
| Piperacillin +/- tazobactam | Broad spectrum | Oral |
| | Broad spectrum | Intravenous |
| Ticarcillin + clavulanate | Broad spectrum | Oral |
| | Broad spectrum | Intravenous |
| ***Cephaloporins (n=0)*** | | |
| Cefazolin | Narrow spectrum | Intravenous |
| Cefuroxime | Broad spectrum | Oral |
| | Broad spectrum | Intravenous |
| Ceftibuten | Broad spectrum | Oral |
| Ceftazidime | Broad spectrum | Intravenous |
| Ceftriaxone | Broad spectrum | Intravenous |
| | Broad spectrum | Intramuscular |
| Cefotaxime | Broad spectrum | Intravenous |
| Cefepime | Broad spectrum | Intravenous |
| Cefaclor | Broad spectrum | Oral |
| Cefoperazone + Sulbactam | Broad spectrum | Intravenous |
| Cefoxitin | Broad spectrum | Intravenous |
| Ceftaroline | Broad spectrum | Intravenous |
| ***Macrolides (n=1*)*** | | |
| Azithromycin | Narrow spectrum | Oral |
| | Narrow spectrum | Intravenous |
| Erythromycin | Narrow spectrum | Oral |
| | Narrow spectrum | Intravenous |
| Clarithromycin | Narrow spectrum | Oral |
| | Narrow spectrum | Intravenous |
| ***Carbapenems (n=0)*** | | |
| Imipenem + cilastatin sodium | Broad spectrum | Intravenous |
| Ertapenem | Broad spectrum | Intravenous |
| Meropenem | Broad spectrum | Intravenous |
| ***Quinolones (n=1)*** | | |
| Ciprofloxacin | Broad spectrum | Oral |
| | Broad spectrum | Intravenous |
| Levofloxacin | Broad spectrum | Oral |
| | Broad spectrum | Intravenous |
| Moxifloxacin | Broad spectrum | Oral |
| | Broad spectrum | Intravenous |

| | | |
|---|---|---|
| ***Tetracyclines (n=1)*** | | |
| Tetracycline | Broad spectrum | Oral |
| | Broad spectrum | Intravenous |
| Doxycycline | Broad spectrum | Oral |
| | Broad spectrum | Intravenous |
| Minocycline | Broad spectrum | Oral |
| | Broad spectrum | Intravenous |
| Tigecyclines | Broad spectrum | Intravenous |
| Oxytetracycline | Broad spectrum | Oral |
| | Broad spectrum | Intravenous |
| | Broad spectrum | Intramuscular |
| ***Aminoglycosides (n=0)*** | | |
| Gentamicin | Broad spectrum | Intravenous |
| Amikacin | Broad spectrum | Intravenous |
| Neomycin | Broad spectrum | Oral |
| Tobramycin | Broad spectrum | Oral |
| | Broad spectrum | Intravenous |
| Streptomycin | Narrow spectrum | Intravenous |
| ***Nitroimidazoles (n=4*)*** | | |
| Metronidazole | Broad spectrum | Oral |
| | Broad spectrum | Intravenous |
| Tinidazole | Broad spectrum | Oral |
| ***Glycopeptides (n=0)*** | | |
| Vancomycin | Narrow spectrum | Oral |
| | Narrow spectrum | Intravenous |
| Teicoplanin | Narrow spectrum | Intravenous |
| ***Others*** | | |
| ***Septrin (n=0)*** | Broad spectrum | Oral |
| | Broad spectrum | Intravenous |
| ***Nitrofurantoin (n=0)*** | Narrow spectrum | Oral |
| ***Rifampicin (n=0)*** | Broad spectrum | Oral |
| | Broad spectrum | Intravenous |
| ***Rifaximin (n=0)*** | Broad spectrum | Oral |

**n = number of respective abx user among BNT recipients**

*** 1 of the clarithromycin user and 4 of the metronidazole user were using combined antibiotics regime with penicillin group**

**Table S2. Adjusted odd ratios of seroconversion of neutralizing antibody among BNT162b2 recipients with different definitions of antibiotic use**

| | Number of antibiotic users according to the definition | Adjusted OR* | 95% CI | p-value |
|---|---|---|---|---|
| *Definition of antibiotic use (by varying duration of usage within six months before first dose of vaccination)* | | | | |
| At least one day (primary analysis) | 29 | 0.26 | 0.08 – 0.96 | 0.032 |
| At least one week (sensitivity analysis) | 26 | 0.31 | 0.09 – 1.30 | 0.083 |
| At least one month (sensitivity analysis) | 1 | 1.12 | NA | 0.990 |
| *Definition of antibiotic use (by varying the time of last antibiotic usage before first dose of vaccination)* | | | | |
| Within six months (primary analysis) | 29 | 0.26 | 0.08 – 0.96 | 0.032 |
| Within three months (sensitivity analysis) | 16 | 0.32 | 0.06 – 2.44 | 0.202 |
| Within one month (sensitivity | 7 | NA | NA | 0.988 |

| | | | | |
|---|---|---|---|---|
| analysis) | | | | |
| Within two weeks (sensitivity analysis) | 4 | NA | NA | 0.992 |
| Within one week (sensitivity analysis) | 3 | NA | NA | 0.991 |

* Adjusted for age ≥ 60 years, male sex, diabetes mellitus, overweight/obesity, hypertension, raised low density lipoprotein (≥3.4 mmol/L), smoking, alcohol use, moderate/severe hepatic steatosis

**Table S3. Adverse reactions after either dose of BNT162b2**

| | **BNT162b2 vaccine recipients** | | |
|---|---|---|---|
| | **Antibiotic users (n =29)** | **Antibiotic non-users (n = 287)** | **p-value** |
| *Total reactions within 7 days after each injection* | | | |
| Any | 25 (86.2%) | 255 (88.9%) | 0.669 |
| Grade 3 or above* | 0 (0) | 0 (0) | 1.000 |
| *Injection site adverse reactions* | 24 (82.8%) | 248 (86.4%) | 0.588 |
| Pain | 22 (75.9%) | 246 (85.7%) | 0.159 |

| | | | |
|---|---|---|---|
| Redness | 3 (10.3%) | 43 (15.0%) | 0.500 |
| Swelling | 9 (31.0%) | 71 (24.7%) | 0.457 |
| Itch | 5 (17.2%) | 26 (9.1%) | 0.158 |
| | | | |
| *Systemic adverse reactions* | 16 (55.2%) | 179 (62.4%) | 0.447 |
| Fever | 6 (20.7%) | 49 (17.1%) | 0.624 |
| Chills and rigors | 3 (10.3%) | 37 (12.9%) | 0.694 |
| Muscle pain | 10 (34.5%) | 101 (35.2%) | 0.939 |
| Joint pain | 4 (13.8%) | 34 (11.8%) | 0.759 |
| Headache | 12 (41.4%) | 77 (26.8%) | 0.097 |
| Fatigue | 12 (41.4%) | 138 (48.1%) | 0.491 |
| Nausea | 1 (3.4%) | 21 (7.3%) | 0.435 |
| Vomiting | 2 (6.9%) | 4 (1.4%) | 0.039 |
| Diarrhea | 2 (6.9%) | 24 (8.4%) | 0.784 |
| Skin rash | 1 (3.4%) | 10 (3.5%) | 0.992 |

*Grade 3 or above: severe or life threatening reactions
